# Perspective on Advancing Health Equity: Enhancing Impact Through Collaboration

**DOI:** 10.1016/j.jacadv.2024.100964

**Published:** 2024-05-08

**Authors:** Alison G.M. Brown, Rebecca Campo, Michelle Freemer, Maliha Ilias, Patrice Desvigne-Nickens, Nicole Redmond, Debbie Vitalis, Charlotte A. Pratt

**Affiliations:** a Division of Cardiovascular Health, National Heart, Lung, and Blood Institute, National Institutes of Health, Bethesda, Maryland, USA; b Division of Lung Diseases, National Heart, Lung, and Blood Institute, National Institutes of Health, Bethesda, Maryland, USA; c Center for Translation Research and Implementation Science, National Heart Lung and Blood Institute, National Institutes of Health, Bethesda, Maryland, USA

**Keywords:** health equity, social determinants of health, structural determinants of health

Despite advances in cardiovascular medicine, disparities in cardiovascular disease persist in the United States by race, ethnicity, socioeconomic status, gender, and geography.^1^ There is a growing recognition that social and structural determinants of health (SDOH) are root causes of health disparities.^1^ Adverse SDOH across domains including housing, education, income and wealth, and access to care, not only impact cardiovascular disease outcomes but also the prevention, treatment, and management of several chronic conditions, including obesity, diabetes, stroke, asthma, and mental health outcomes.^2^ Given the complexity of the root causes of health disparities, achieving equity in cardiovascular care will require innovative collaboration across sectors, specializations, and disciplines that transcend diseases and conditions. This Viewpoint calls for multidisciplinary and multisectoral collaborations in research and clinical care to address SDOH, highlighting innovative examples of health equity research initiatives and community engagement supported by the National Heart, Lung, and Blood Institute (NHLBI) and broadly across National Institutes of Health (NIH).

Social and structural determinants of health include educational and economic opportunities, neighborhood environment (including access to green space, walkable neighborhoods, and healthy foods), food security, access to and quality of health care systems, as well as community and social context which impact the individual and community risks and protective factors.^3^ Additionally, the chronic stress of daily coping with social disadvantages due to adverse SDOH can impact health outcomes directly and indirectly. From a lifecourse perspective, SDOH can also add to health disparities based on the timing of exposure (eg, pregnancy and early childhood) as well as through the cumulative effect of exposures throughout the lifespan and intergenerationally.^4^ Therefore, health equity research should not only consider the various biological, behavioral, and sociocultural factors at the individual level over the lifespan but also address health disparities at the organizational, community, and societal levels.

The call for multisectoral and multidisciplinary approaches in health care and research recognizes that complex problems require multifaceted, nuanced solutions which integrate diverse perspectives through strategic collaborations. Research consistently shows that diversity in teams lends itself to better outcomes and greater innovation. To adequately address the structural and social challenges that communities and patients face in accessing health care and managing disease, multiple sectors need to be engaged in designing sustainable solutions that address the root causes of disease. These sectors range from medicine, technology, education, housing, and transportation to commerce, agriculture, economic and urban development, and human and social services.

Meaningful collaboration of key players in the health care system is paramount in dissemination and implementation science to improve the adaptation and uptake of evidenced-based interventions in the communities that need them most.^5^ For example, to adequately address barriers and facilitators for the uptake of evidence-based clinical practice, multiple perspectives need to be considered including clinicians, patients, and the allied health professional required to execute and sustain the interventions (eg, social workers, nurses, physician assistants, dietitians, and medical billing staff). Patient-centered team-based care, or what the World Health Organization terms “interprofessional collaborative practices,” include multiple health care professionals collaborating with the patient and their families to deliver high-quality patient care.^6^ These approaches have been associated with better health outcomes and patient satisfaction, and lower health care utilization for patients with comorbid conditions and complex medical needs.^7^^,^^8^

For community-based health disparities research, collaboration with communities and those with lived experiences is integral, particularly when working with historically disenfranchised communities with low levels of trust in the biomedical research enterprise. Meaningful engagement with multiple partners in the community needs to occur at every stage of the research process, from concept to dissemination of research findings, and should extend beyond consultation to shared decision-making and leadership.^9^ Equitable and meaningful collaboration with key community partners helps build trust and shared values around science and research and is essential for program sustainability.^10^

Since the 1985 Secretary Heckler’s Report on *Black and Minority Health*, NIH has been committed to eliminating health disparities. Recent efforts to advance health equity at NIH and NHLBI address SDOH factors by fostering collaborations to address these challenges. Highlighted below are NHLBI-supported Notice of Funding Opportunities (NOFOs), Notice of Special Interests, and workshops that have sought to stimulate science in this area and encourage equitable collaborations across multidisciplinary research teams, including federal partners and community members throughout the research spectrum. Table 1 includes additional examples and workshop executive summaries highlighting research opportunities to curtail health disparities and promote health equity.

**Table 1:** Examples of NHLBI-Supported Workshops and NIH-Wide Initiatives That Encourage Multisectoral and Multidisciplinary Research

Initiative, Program, or Workshop Title	Links to Executive Summaries or Initiatives
Active initiatives^a^	
Data Informed, Place-Based Community-Engaged Research to Advance Health Equity	https://grants.nih.gov/grants/guide/notice-files/NOT-HL-23-110.html
Multi-sectoral Preventive Interventions that Address Social Determinants of Health in Populations that Experience Health Disparities	https://grants.nih.gov/grants/guide/pa-files/PAR-24-053.html
Transformative Research to Address Health Disparities and Advance Health Equity	https://grants.nih.gov/grants/guide/rfa-files/RFA-NR-24-004.html
Stimulating Research to Understand and Address Hunger, Food, and Nutrition Insecurity	https://grants.nih.gov/grants/guide/notice-files/NOT-OD-22-135.html
ADVANCE Predoctoral T32 Training Program to Promote Diversity in Health Disparities Research, Preventive Interventions, and Methodology	https://grants.nih.gov/grants/guide/rfa-files/RFA-OD-23-018.html
Leveraging Existing and Accessible Datasets for Implementation Research Strategies and Testing - LEAD FIRST	https://grants.nih.gov/grants/guide/notice-files/NOT-HL-23-120.html
Launched Programs	
Early Intervention to Promote Cardiovascular Health of Mothers and Children (ENRICH)	https://grants.nih.gov/grants/guide/rfa-files/RFA-HL-22-007.html
Implementing a Maternal health and PRegnancy Outcomes Vision for Everyone (IMPROVE)	https://www.nichd.nih.gov/research/supported/IMPROVE/about
Disparities Elimination through Coordinated Interventions to Prevent and Control Heart and Lung Disease Risk (DECIPHeR)	https://grants.nih.gov/grants/guide/rfa-files/RFA-HL-20-003.html
Community Engagement Alliance (CEAL)	https://ceal.nih.gov/
Workshops	
Food Insecurity, Neighborhood Food Environment, and Nutrition Health Disparities: State of the Science	https://www.nhlbi.nih.gov/events/2021/food-insecurity-neighborhood-food-environment-and-nutrition-health-disparities
Pregnancy and Maternal Conditions that Increase Risk of Morbidity and Mortality	https://www.nhlbi.nih.gov/events/2020/maternal-mortality-workshop
The Science of Precision Prevention to Reduce Disparities in Cardiovascular Health	https://www.nhlbi.nih.gov/events/2022/science-precision-prevention-reduce-disparities-cardiovascular-health
Advancing Health Equity Through Culture-Centered Dietary Interventions to Address Chronic Diseases	https://www.nhlbi.nih.gov/events/2023/advancing-health-equity-through-culture-centered-dietary-interventions
Workshop on Housing and Obesity: Gaps, Opportunities, and Future Directions for Advancing Health Equity	https://www.niddk.nih.gov/news/meetings-workshops/2022/workshop-housing-obesity
Advancing the Science of Community-Engaged Health Disparities Research	https://www.nhlbi.nih.gov/events/2022/advancing-science-community-engaged-health-disparities-research
Centering Communities in Dissemination Research to Promote Health Equity	https://www.nhlbi.nih.gov/events/2023/centering-communities-dissemination-research-promote-health-equity
Enhancing Implementation of Guidelines and Evidence-based Care in Clinical and Community Settings (ENGAGE) Workshop	https://www.nhlbi.nih.gov/events/2023/engage_workshop
Exploring Research Opportunities at the Intersection of Planetary and Cardiovascular Health Virtual Workshop	https://www.nhlbi.nih.gov/events/2023/exploring-research-opportunities-intersection-planetary-and-cardiovascular-health

NHLBI = National Heart, Lung, and Blood Institute; NIH = National Institutes of Health.

a Active at time of publication. Please check NHLBI funding opportunities for expiration date.

To address the disparities in maternal morbidity and mortality in the United States, NHLBI is supporting the “*Early Intervention to Promote Cardiovascular Health of Mothers and Children*” (ENRICH) Program. As an example of a federal partnership with community organizations, NHLBI is collaborating with other NIH Institutes, Centers, and Offices, the Administration for Children and Families, Health Resources and Services Administration, and local and national evidence-based home visiting organizations to implement a multicenter trial to promote cardiovascular health of mothers and children of low socioeconomic status, or who live in low resource geographic areas.

Place-based structural factors are another large inequity requiring interdisciplinary collaborations and community partnerships. Recognizing this, NHLBI led an NIH Notice of Special Interest “*Data Informed, Place-Based Community-Engaged Research to Advance Health Equity*” (NOT-HL-23-110) that requires collaboration among multidisciplinary research teams (eg, data scientists, geographers, health services researchers, etc) to develop community-engaged research armed with geospatial data to inform development of interventions to address geographic factors in health inequities.

Given the intransigence of disparities in pediatric asthma, despite some evidence-based interventions to improve outcomes, federal agencies collaborated to identify a formal plan to maximize areas of synergy among their agencies’ efforts (*Coordinated Federal Action Plan to Reduce Racial and Ethnic Asthma Disparities*). As a direct result of that collective effort, NHLBI supported funding announcements “*Creating Asthma Empowerment Collaborations to Reduce Childhood Asthma Disparities*” (RFA-HL15-028) and “*Asthma Empowerment Collaborations to Reduce Childhood Asthma Disparities*” (RFA HL17-001) requiring investigators to develop multisector, evidence-based interventions to meet the needs of their respective communities, leveraging available federal resources and recognizing the need to support children with asthma and their families beyond the provision of medical care.

With the goal to promote health equity research and reduce health disparities, the “Community Partnerships to Advance Science for Society” (ComPASS) Program is an NIH Common Fund effort for structural intervention research to improve health outcomes in communities experiencing health disparities. Community organizations and researchers are required to collaborate as equal partners along the research spectrum to support community-led, structural interventions that consists of changing the social, physical, economic, or political environments. In another example, the NIH-wide “*Community Engagement Alliance*” (CEAL) was developed as part of the Federal government’s response to the COVID-19 pandemic by encouraging meaningful community engagement to address the health disparities highlighted during the pandemic, build trust around science, and increase inclusive participation in clinical research.^10^ The CEAL Alliance has expanded its focus to address health disparities in maternal health, climate health, health knowledge, primary care research, and also provides technical assistance and learning opportunities to support community engagement and community-engaged research.

Multisectoral collaborations across service sectors and community-based organizations are critical to address SDOH and promote health equity. Another NHLBI supported effort includes the NIH Office of Disease Prevention *Advancing Prevention Research for Health Equity* (ADVANCE) initiative and its NIH-wide NOFO “*Multi-sectoral Preventive Interventions that Address Social Determinants of Health in Populations that Experience Health Disparities*” (PAR-24-053) that calls for multisectoral preventive interventions (ie, 2 or more service sectors, such as public health, education, housing, parks, and recreation). In addition, “*The Transformative Research to Address Health Disparities and Advance Health Equity*” (RFA-NR-24-004) is an NIH-wide NOFO that seeks to advance innovative interventions, strategies, policies, programs, and environmental changes to address health disparities. Specifically, the initiative calls for the development and testing of interventions in a variety of U.S. settings (eg, child welfare, criminal justice), dissemination and implementation research that addresses structural SDoH factors and conditions of daily life (eg, beyond individual/family level), or evaluations of U.S. policy, programmatic, or environmental changes relevant to prevent, reduce, or eliminate health disparities. Multisectoral and community partnerships are required (eg, housing, transportation, food system sectors, faith-based organizations) and should include meaningful engagement such as co-creation of research objectives, bidirectional communication and shared leadership and decision-making. The NHLBI’s “*Disparities Elimination through Coordinated Interventions to Prevent and Control Heart and Lung Disease Risk*” (DECIPHeR) Alliance is a community-engaged implementation research program that is addressing heart and lung health in U.S. communities through multisectoral teams.

The NHLBI also convenes workshops in collaboration with federal partners and extramural investigators that encourage multidisciplinary, multisectoral health equity research. For example, the workshop “*Advancing Health Equity Through Culture Centered Dietary Interventions to Address Chronic Disease*” was held in 2023 in collaboration with NIH Institutes, Centers, and Offices, the Centers for Disease Control and Prevention, the Indian Health Service, Health and Human Services Office of the Assistant Secretary for Health, and the U.S. Department of Agriculture, with discussions highlighting the need for interdisciplinary collaboration, community-centered approaches, and considerations for structural/economic factors. In December 2023, NHLBI held a workshop on the intersection of planetary and cardiovascular health involving transdisciplinary experts from environmental health, urban planning, social and behavioral sciences, engineering, and public and cardiovascular health. Additionally, the 2022 workshop on “*Advancing the Science of Community-Engaged Health Disparities Research*” convened a variety of key players in the field, including researchers, health care providers, community planners and engineers, and federal, state, and local government officials who discussed the current state and future directions of community-engaged health disparities research both in the U.S. and internationally.

In conclusion, achieving health equity, through research or clinical care, requires moving beyond siloes and collaborating across sectors, disciplines, and areas of specialization. For NHLBI, this includes enhancing partnerships among federal or local governments, encouraging multisectoral research that targets levels beyond the individual, and family, with collaborative multidisciplinary teams that transcend research institutional boundaries and, most importantly, engaging the community as true partners throughout the research process.

## Funding support and author disclosures

The authors have reported that they have no relationships relevant to the contents of this paper to disclose.
